# Factors Associated with Short-Term Clinical Outcomes in ICU Patients with Carbapenem-Resistant *Acinetobacter baumannii* Pneumonia: A Single-Center Retrospective Cohort Study

**DOI:** 10.3390/jcm15103594

**Published:** 2026-05-08

**Authors:** Yong Hoon Lee, Ji Eun Park, Hyewon Seo, Jaehee Lee, Won Kee Lee, Sun Ha Choi, Seung Soo Yoo, Shin Yup Lee, Seung-Ick Cha, Chang Ho Kim

**Affiliations:** 1 Department of Internal Medicine, School of Medicine, Kyungpook National University, 680 Gukchaebosang-ro, Jung-gu, Daegu 41944, Republic of Korea; jieun@knu.ac.kr (J.E.P.); seohw@knu.ac.kr (H.S.); jaelee@knu.ac.kr (J.L.); sunha20@knu.ac.kr (S.H.C.); yooss@knu.ac.kr (S.S.Y.); shinyup@knu.ac.kr (S.Y.L.); sicha@knu.ac.kr (S.-I.C.); kimch@knu.ac.kr (C.H.K.); 2 Biostatistics, Medical Research Collaboration Center, Kyungpook National University, Daegu 41944, Republic of Korea; wonlee@knu.ac.kr

**Keywords:** *Acinetobacter baumannii*, carbapenem resistance, clinical outcomes, pneumonia, intensive care units

## Abstract

**Background:** Carbapenem-resistant *Acinetobacter baumannii* (CRAB) infections carry a high mortality rate, yet effective treatments remain scarce. CRAB infections have become increasingly common in intensive care units (ICUs), but data specifically examining CRAB pneumonia in ICU patients remain limited. This study aimed to identify factors associated with in-hospital mortality and day-14 clinical failure in ICU patients with CRAB pneumonia. **Methods:** Data from 173 ICU patients with CRAB pneumonia admitted to a tertiary referral hospital in the Republic of Korea between January 2018 and June 2025 were retrospectively analyzed. Multivariable logistic regression was used to identify factors independently associated with in-hospital mortality and day-14 clinical failure. **Results:** The median patient age was 70 years, and ventilator-associated pneumonia accounted for 76.3% of cases. In-hospital mortality reached 80.9% (140/173), and 75.7% (131/173) experienced clinical failure at day 14. After adjustment, higher serum albumin was associated with lower in-hospital mortality (odds ratio [OR], 0.55; 95% confidence interval [CI], 0.31–0.95; *p* = 0.031), whereas corticosteroid use was associated with higher mortality (OR, 2.82; 95% CI, 1.23–6.50; *p* = 0.015). However, in an exploratory dose-based analysis among patients who received corticosteroids, prednisone-equivalent dose was not significantly associated with in-hospital mortality. For day-14 clinical failure, higher C-reactive protein and higher Sequential Organ Failure Assessment scores were associated with increased failure, while minocycline use was associated with reduced failure (OR, 0.21; CI, 0.08–0.50; *p* = 0.001). **Conclusions:** Lower albumin levels and corticosteroid use were independently associated with higher in-hospital mortality among ICU patients with CRAB pneumonia. Minocycline may offer clinical benefit, although further validation is needed.

## 1. Introduction

Carbapenem-resistant *Acinetobacter baumannii* (CRAB) is a major cause of life-threatening nosocomial infections [1]. Treatment options remain limited because the organism possesses both inherent and acquired resistance mechanisms [1]. CRAB infections carry high mortality and have increased in prevalence worldwide, creating a substantial public health concern [2]. A systematic analysis estimated that CRAB was responsible for 57,700 attributable deaths worldwide in 2019 [3]. Mortality is particularly high in bloodstream infection and hospital-acquired/ventilator-associated pneumonia, with reported mortality rates of 42% in bacteremia and a pooled mortality of 42.6% in multidrug-resistant A. baumannii lung infections [4,5]. Infection control measures designed to prevent CRAB transmission and outbreaks also impose significant operational challenges for healthcare facilities [6]. Reflecting this urgency, the World Health Organization designates CRAB as a “critical-priority” pathogen and calls for accelerated research and development of effective therapies [7].

*Acinetobacter baumannii* frequently infects the respiratory tract and is a common cause of pneumonia, particularly among patients in intensive care units (ICUs) who require mechanical ventilation [8]. The proportion of *A. baumannii* isolates resistant to carbapenems continues to rise, and CRAB now accounts for a substantial share of carbapenem-resistant Gram-negative pathogens in ICU settings [9,10]. CRAB pneumonia is associated with higher mortality than pneumonia caused by carbapenem-susceptible *A. baumannii* [11].

Although prior studies have addressed CRAB infection in critically ill patients, data specifically evaluating prognostic factors for short-term clinical outcomes in ICU patients with CRAB pneumonia remain limited [12,13]. Therefore, in this retrospective cohort study, we aimed to identify factors associated with in-hospital mortality and day-14 clinical failure in this population.

## 2. Methods

### 2.1. Study Design

This retrospective cohort study was conducted at Kyungpook National University Hospital (KNUH), a tertiary referral center in Daegu, Republic of Korea. Clinical data were retrospectively extracted from the electronic medical records of consecutive patients admitted to the ICUs between 1 January 2018, and 30 June 2025. For the primary analysis, patients were classified as survivors or non-survivors according to in-hospital mortality, and their clinical characteristics were compared between groups. Day-14 clinical failure was evaluated as a secondary outcome. This study was reported in accordance with the Strengthening the Reporting of Observational Studies in Epidemiology (STROBE) guideline.

### 2.2. Ethical Considerations

The study protocol was approved by the Institutional Review Board of Kyungpook National University Hospital (KNUH 2026-01-006; approved on 9 February 2026). The requirement for informed consent was waived because of the retrospective design of the study. Patient data were de-identified prior to analysis.

### 2.3. Patient Selection

Patients with CRAB isolated from respiratory specimens were assessed for eligibility according to the prespecified study definition of CRAB pneumonia, based on predefined clinical and radiologic criteria. Two investigators reviewed study eligibility, and cases with uncertainty or disagreement were further discussed with the corresponding author until consensus was reached. Only patients who survived for at least 48 h after collection of the index specimen were included. Exclusion criteria were age <18 years, inadequate respiratory specimens, and a pre-existing do-not-resuscitate order.

### 2.4. Definitions

CRAB pneumonia was defined as a positive respiratory culture for CRAB, accompanied by clinical and radiologic features consistent with pneumonia. Radiologic criteria required new or progressive lung infiltrates on chest radiography. Clinical criteria required at least two of the following: (1) body temperature > 38.0 °C or <36.0 °C, (2) leukocytosis (≥12,000/mm^3^) or leukopenia (≤4000/mm^3^), and (3) macroscopically purulent tracheal aspirate or sputum. These clinical criteria were adapted from established clinical criteria for suspected hospital-acquired/ventilator-associated pneumonia [14]. Respiratory specimens were considered adequate if they met the following quality criteria: (1) quantitative bronchoalveolar lavage culture ≥1 × 10^4^ colony-forming units (CFU)/mL; (2) quantitative bronchoscopic or endotracheal aspirate culture ≥1 × 10^5^ CFU/mL; (3) semi-quantitative endotracheal aspirate culture showing moderate or greater growth; or (4) sputum containing <10 squamous epithelial cells per low-power field. Ventilator-associated pneumonia (VAP) was defined as pneumonia occurring at least 48 h after mechanical ventilation [15].

Immunosuppression was defined as the presence of at least one of the following: neutropenia (absolute neutrophil count or total white blood cell count <500/mm^3^), hematologic malignancy, human immunodeficiency virus infection with CD4 count <200, prior splenectomy, solid organ or hematopoietic stem cell transplant, cytotoxic chemotherapy, immunosuppressant use, or daily systemic corticosteroid therapy at a prednisone-equivalent dose of ≥20 mg/day for ≥14 days) [16,17]. Polymicrobial infection was defined as the detection of at least one additional bacterial species from clinical specimens obtained on the index culture date or within 7 days thereafter. Clinical failure was defined as persistent or worsening signs or symptoms of pneumonia without radiologic improvement, or death from any cause. In this retrospective study, this outcome was assessed based on the overall clinical course documented in the medical records, including vital signs, laboratory findings, respiratory status, chest radiographic findings, and physician documentation.

### 2.5. Data Collection

Clinical data were retrospectively extracted from the electronic medical records using predefined study variables. Extracted data included baseline demographic characteristics, comorbidities, laboratory results, and ICU admission variables, including Acute Physiology and Chronic Health Evaluation (APACHE) II and Sequential Organ Failure Assessment (SOFA) scores.

For each CRAB pneumonia case, we determined whether the episode represented VAP and recorded the SOFA score on the index date (the day the index culture was collected). We also documented the presence of polymicrobial infection and whether CRAB was concurrently isolated from normally sterile sites, such as blood, abdominal fluid, pleural fluid, or cerebrospinal fluid.

Corticosteroid use was defined as receipt of at least one dose equivalent to ≥10 mg prednisone within 2 weeks of the index date. This cutoff was used as an operational definition to capture any systemic corticosteroid exposure across heterogeneous regimens in this retrospective cohort. Among patients who received corticosteroids within this time window, the prednisone-equivalent corticosteroid dose (mg/day) was recorded as the first documented dose. Antibiotic use—including meropenem, minocycline, and colistin—was recorded when administered for at least 2 days within the same period. Combination therapy was defined as concomitant use of two or more of these antibiotics for at least 2 days. ICU interventions, including invasive mechanical ventilation, initiation of renal replacement therapy, and pleural drainage via chest tube or percutaneous catheter, were recorded.

### 2.6. Outcomes

The primary outcome of this study was in-hospital mortality. The secondary outcome was day-14 clinical failure, which was defined as persistent or worsening signs or symptoms of pneumonia without radiologic improvement, or death from any cause. Day-14 clinical failure was assessed based on the overall clinical course documented in the medical records, including vital signs, laboratory findings, respiratory status, chest radiographic findings, and physician documentation.

### 2.7. Statistical Analysis

Continuous variables were summarized as medians with interquartile ranges (IQRs), and categorical variables were summarized as counts with percentages. Group comparisons used Student’s *t* test when normality assumptions were met and the Mann–Whitney U test otherwise. Categorical variables were compared using the chi-square test or Fisher’s exact test, as appropriate. To identify independent factors associated with clinical outcomes, we performed multivariable logistic regression. In regression analyses, continuous variables were entered as continuous terms (not categorized), and odds ratios with 95% confidence intervals (CIs) were reported per 1-unit increase (e.g., per 1 g/dL increase in serum albumin). Variables with *p* < 0.05 in univariable analyses, as well as variables considered clinically relevant, were entered into the multivariable models. Independent predictors were identified using backward elimination. Before final model selection, multicollinearity among candidate covariates was assessed using variance inflation factors (VIFs). Missing data were limited to several baseline laboratory variables, and variable-specific denominators are provided where applicable. Given minimal missingness, no imputation was performed. Statistical analyses were performed using R software (version 4.4.2; R Foundation for Statistical Computing, Vienna, Austria). A two-sided *p* value < 0.05 was considered statistically significant.

### 2.8. Use of AI-Assisted Language Editing

The initial draft of the manuscript was prepared by the authors. ChatGPT (OpenAI, San Francisco, CA, USA) was used only for English-language polishing and improving readability. ChatGPT was not used for study design, data collection, data analysis, interpretation of results, or generation of scientific conclusions. All AI-assisted text was reviewed and edited by the authors, who take full responsibility for the content of the manuscript.

## 3. Results

**Figure 1:**
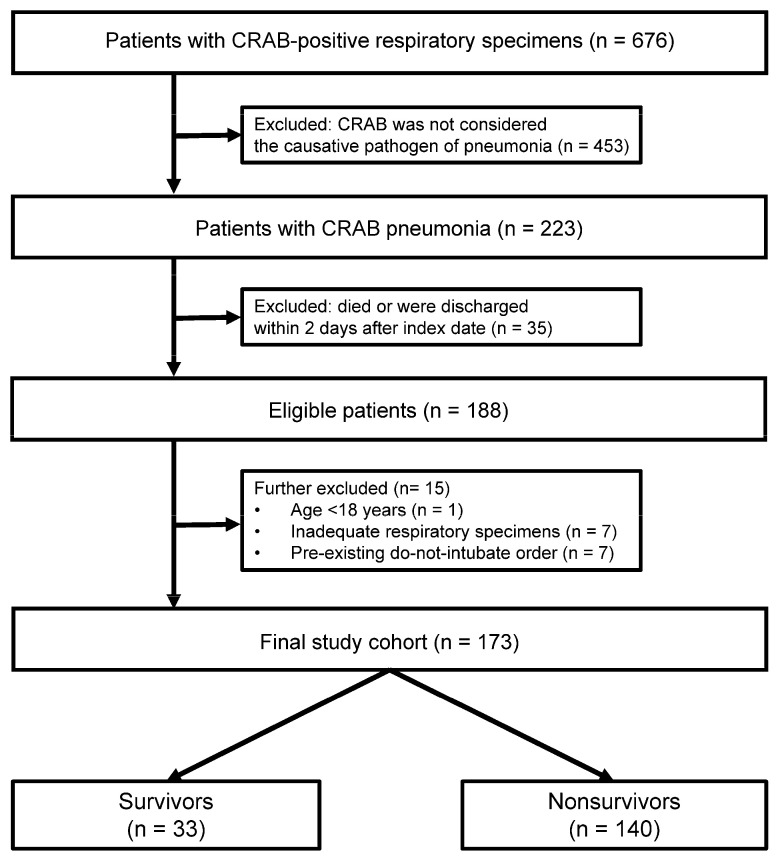
Flowchart of patient selection. CRAB, Carbapenem-Resistant *Acinetobacter baumannii*.

We initially identified 676 patients with at least one respiratory specimen positive for CRAB. Of these, 453 were excluded because the available clinical, radiologic, and microbiologic data did not support CRAB as the causative pathogen of pneumonia. An additional 35 patients with presumed CRAB pneumonia were excluded because they could not be observed for at least 2 days after the index date. Among the remaining 188 patients, we further excluded 1 patient younger than 18 years, 7 patients with inadequate respiratory specimens, and 7 patients with a pre-existing do-not-intubate order. A total of 173 patients were therefore included in the final analysis (Figure 1). Among these 173 patients, 140 (80.9%) died during hospitalization. Clinical failure at day 14 was observed in 131 patients (75.7%). The median follow-up time from the index date to hospital discharge or death was 13 days (IQR, 6–27).

### 3.1. Baseline Characteristics

Baseline demographic and clinical characteristics are summarized in Table 1. The median age of the cohort was 70 years, and 69.4% of patients were male. Most patients were admitted to the medical ICU (71.7%), and 64.2% required mechanical ventilation at ICU admission. Comorbid conditions did not differ significantly between survivors and non-survivors. At ICU admission, APACHE II and SOFA scores tended to be higher in non-survivors than in survivors, although these differences were not statistically significant.

Among initial laboratory findings (Table 2), non-survivors had significantly higher C-reactive protein (CRP) levels and lower serum albumin levels than survivors (CRP: 7.5 mg/dL [0.4–18.4] vs. 0.8 mg/dL [0.1–3.2], *p* = 0.004; albumin: 3.0 g/dL [2.6–3.6] vs. 3.5 g/dL [3.2–4.0], *p* = 0.001).

### 3.2. CRAB Pneumonia and Treatment Details

Variables related to CRAB pneumonia and its treatment are presented in Table 3. The SOFA score on the index date was significantly higher in non-survivors than in survivors (8 [5–10] vs. 6 [4–7], *p* = 0.001). Corticosteroid use was also significantly more frequent among non-survivors (93/140 [66.4%] vs. 14/33 [42.4%], *p* = 0.019). Minocycline use tended to be more common in survivors, although it did not reach statistical significance (*p* = 0.060). Use of other antibiotics did not differ significantly between groups. New-onset renal replacement therapy (RRT) during the ICU stay occurred significantly more often in non-survivors than in survivors (39/140 [27.9%] vs. 2/33 [6.1%], *p* = 0.015).

### 3.3. Factors Associated with Clinical Outcomes

**Figure 2:**
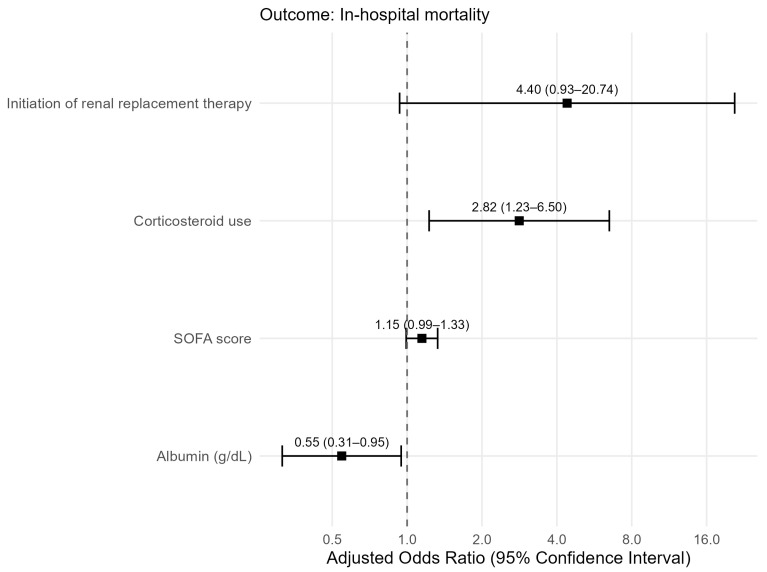
Forest plot of factors associated with in-hospital death in multivariable logistic regression. SOFA, Sequential Organ Failure Assessment.

Multivariable logistic regression included age, APACHE II score, CRP, serum albumin, SOFA score on the index date, corticosteroid use, minocycline use, and new-onset RRT (Table 4). No missing values were observed for the variables included in the multivariable model. No substantial multicollinearity was identified among the candidate covariates, with all VIFs < 1.5. Higher serum albumin levels were independently associated with a reduced risk of in-hospital mortality (odds ratio [OR] 0.55; 95% CI, 0.31–0.95; *p* = 0.031), whereas corticosteroid use was independently associated with increased mortality (OR 2.82; 95% CI, 1.23–6.50; *p* = 0.015). The corresponding forest plot is shown in Figure 2.

A sensitivity analysis that included patients with pre-existing do-not-resuscitate (DNR) orders (total *n* = 180) and adjusted for DNR status produced similar results. Higher serum albumin levels (OR 0.55; 95% CI, 0.32–0.95; *p* = 0.030) and corticosteroid use (OR 2.60; 95% CI, 1.16–5.83; *p* = 0.021) remained significant. Full multivariable results are provided in Supplementary Table S1.

In a subgroup analysis of the 107 patients who received corticosteroids, a multivariable model similar to the primary model was constructed, except that new-onset RRT was excluded because no patients in one outcome group underwent new-onset RRT. Among these patients, the median prednisone-equivalent corticosteroid dose was 50 mg/day (IQR, 33.3–66.7 mg/day). Prednisone-equivalent corticosteroid dose, modeled per 10 mg/day increase, was not significantly associated with in-hospital mortality in the univariate analysis and did not remain in the final subgroup multivariable model after backward elimination. In this subgroup, minocycline use was associated with lower in-hospital mortality (OR 0.19; 95% CI, 0.04–0.90; *p* = 0.036, Supplementary Table S2).

**Figure 3:**
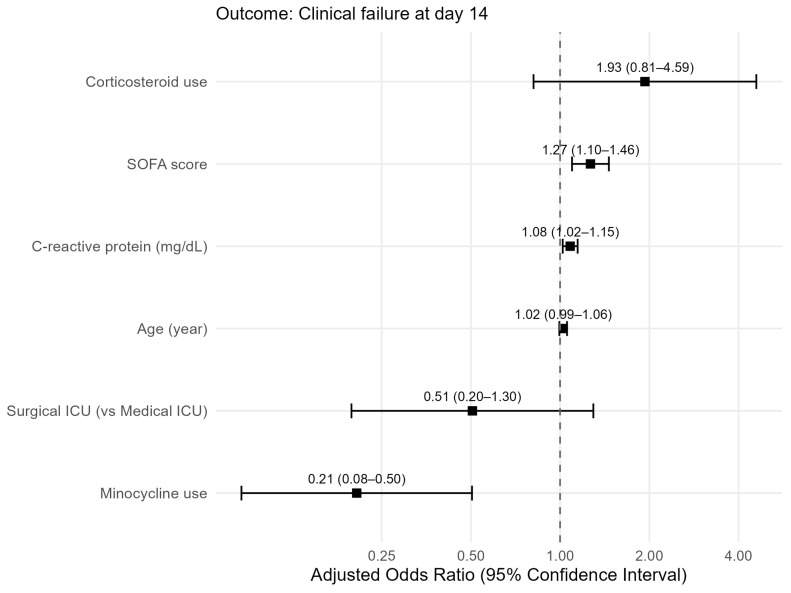
Forest plot of factors associated with day-14 clinical failure in multivariable logistic regression. ICU, intensive care unit; SOFA, Sequential Organ Failure Assessment.

For the secondary outcome of day-14 clinical failure, both univariable and multivariable analyses were performed (Table 5). No substantial multicollinearity was identified among the candidate covariates in this model, with all VIFs < 1.7. Higher CRP levels (OR 1.08; 95% CI, 1.02–1.15; *p* = 0.008) and higher SOFA scores on the index date (OR 1.27; 95% CI, 1.10–1.46; *p* = 0.001) were significantly associated with clinical failure. Minocycline use was associated with a decreased risk of clinical failure (OR 0.21; 95% CI, 0.08–0.50; *p* = 0.001). The forest plot for this analysis is shown in Figure 3.

## 4. Discussion

This study aimed to identify factors associated with in-hospital mortality and day-14 clinical failure in ICU patients with CRAB pneumonia. In this retrospective ICU cohort, in-hospital mortality approached 80%. In adjusted analyses, lower serum albumin levels and corticosteroid use were independently associated with in-hospital mortality. For the secondary outcome, higher SOFA scores and elevated CRP levels were associated with day-14 clinical failure, whereas minocycline use was associated with a lower risk of failure. The associations involving albumin, CRP, and SOFA score are clinically plausible, as these variables may reflect nutritional reserve, inflammatory burden, and overall severity of illness in critically ill patients with CRAB pneumonia. In addition, the observed associations of corticosteroid exposure and minocycline use with clinical outcomes may indicate that treatment-related factors also warrant further investigation, although these findings should be interpreted cautiously given the retrospective observational design. These findings may help identify ICU patients with CRAB pneumonia who are at particularly high risk of poor short-term outcomes and may support closer monitoring and early risk stratification in clinical practice.

The approximately 80% in-hospital mortality observed in our study aligns with prior reports highlighting the substantial mortality burden of CRAB infection in critically ill patients. For example, a study of ICU-acquired CRAB bacteremia reported a 30-day mortality rate of 79.8% [18]. Similarly, in a multicenter retrospective study of 760 patients with CRAB colonization, those who developed pneumonia had an in-hospital mortality rate of 79.6% (312/392), compared with 72.8% (268/368) among those who did not develop pneumonia [19].

Albumin plays multiple physiological roles, including maintaining colloid oncotic pressure, transporting diverse molecules, exerting antioxidant and anti-inflammatory effects, and supporting endothelial integrity [20,21,22]. Serum albumin levels reflect nutritional status and hepatic synthetic function and serve as a biomarker of acute illness severity [23]. Hypoalbuminemia is common in critical illness due to systemic inflammation, capillary leak, fluid shifts, limited nutritional intake, dilutional effects of resuscitation, increased renal or enteric losses, and reduced hepatic synthesis [22,24,25,26]. Prior studies have linked hypoalbuminemia to adverse outcomes in ICU patients [23,27,28,29], but evidence specific to CRAB pneumonia has been limited. Our findings suggest that ICU patients with low albumin—particularly in settings with ongoing CRAB transmission—may require closer monitoring and enhanced infection-prevention strategies. Future research should clarify the prognostic value of serum albumin and determine whether early or targeted albumin supplementation can improve clinical outcomes in ICU patients with CRAB pneumonia.

Corticosteroid use was also independently associated with mortality. Because corticosteroids are often administered as adjunctive therapy in patients with greater illness severity [30], including those with septic shock, this association may reflect unmeasured confounding. Although limited observational data have suggested potential harm from systemic corticosteroids in Gram-negative septic shock [31], other studies in sepsis and pneumonia have reported potential benefits in selected populations [32,33]. However, these studies were conducted in populations that differ from our cohort of critically ill patients with CRAB pneumonia and therefore may not be directly generalizable. In addition, emerging evidence suggests that corticosteroid responsiveness may not be explained by clinical severity alone, but may vary according to underlying biological or immune heterogeneity [34]. Therefore, our finding regarding corticosteroid use should be interpreted cautiously and warrants validation in larger, well-designed studies.

Minocycline use was associated with improved outcomes. Minocycline, a tetracycline derivative with in-vitro activity against CRAB, is considered a therapeutic option for CRAB infections [8,35]. Prior studies have suggested that tetracycline-containing regimens may be effective [36,37,38]. A recent multicenter retrospective cohort study by Seok et al. similarly found that minocycline-based therapy improved outcomes in CRAB pneumonia [39], consistent with our findings. Despite these encouraging signals, establishing minocycline’s effectiveness remains challenging due to small sample sizes, potential confounding factors, and heterogeneity in infection sources. Other therapeutic options, such as sulbactam-containing agents, polymyxins, and cefiderocol, are also used for CRAB infections, but limited clinical data prevent preferential recommendation of any single agent [40]. Moreover, newer agents such as sulbactam–durlobactam are not yet available in the Republic of Korea. Given the scarcity of effective treatments and the high mortality of CRAB pneumonia, our findings support minocycline as a reasonable therapeutic option in ICU settings, though confirmatory studies are needed.

This study has several limitations. First, the retrospective design of this study introduces the possibility of selection bias, misclassification, and unmeasured confounding. Because CRAB is frequently isolated from respiratory specimens as a colonizer in critically ill patients, determining whether CRAB was the true causative pathogen of pneumonia was inherently challenging. Presumed CRAB pneumonia was defined using predefined clinical and radiologic criteria; however, some degree of diagnostic uncertainty remained. In addition, 50 patients (28.9%) in the final cohort had polymicrobial infection, and the contribution of non-CRAB pathogens to clinical outcomes could not be fully excluded. Therefore, the observed associations should be interpreted with caution. Second, although we captured exposure to multiple therapeutic agents, detailed longitudinal dosing information was not consistently available, limiting a robust assessment of dose–response relationships. This issue was particularly relevant to corticosteroid exposure. In the primary analysis, corticosteroid use was defined dichotomously as any systemic corticosteroid dose equivalent to ≥10 mg of prednisone within 2 weeks of the index date, which may not have adequately reflected treatment intensity, duration, or cumulative exposure. Although we additionally performed an exploratory analysis using the prednisolone-equivalent dose on the first day of corticosteroid use within this 14-day window, this measure may still not have fully captured subsequent dose changes or the heterogeneity in treatment timing and indication. Third, the relatively limited sample size may have affected the robustness of the multivariable models. Although no substantial multicollinearity was identified among the candidate variables, the possibility of residual confounding and unstable estimates cannot be excluded. Fourth, limited drug availability in the Republic of Korea (e.g., the lack of intravenous minocycline and newer agents such as cefiderocol) may limit generalizability to settings with broader antimicrobial access. Finally, the single-center design and modest sample size may limit external validity.

## 5. Conclusions

In conclusion, lower albumin levels and corticosteroid use were independently associated with in-hospital mortality among ICU patients with CRAB pneumonia. However, an exploratory dose-based analysis among patients who received corticosteroids did not identify a significant association between prednisone-equivalent dose and mortality. Minocycline may offer clinical benefits; however, further studies are needed to validate this finding and clarify its role in treatment. Larger multicenter studies are also warranted to confirm these associations and improve risk stratification in critically ill patients with CRAB pneumonia.

## Figures and Tables

**Table 1:** Baseline characteristics.

Variable	Total (*n* = 173)	Survivor (*n* = 33)	Non-Survivor (*n* = 140)	*p*-Value
Age	70 (61–77)	66 (62–78)	71 (61–76)	0.395
Male	120 (69.4)	25 (75.8)	95 (67.9)	0.499
Body mass index (kg/m^2^)	22.4 (20.3–24.8)	22.4 (20.4–23.5)	22.4 (20.3–25.2)	0.219
Comorbidity				
Chronic pulmonary disease	20 (11.6)	4 (12.1)	16 (11.4)	>0.999
Hypertension	75 (43.4)	13 (39.4)	62 (44.3)	0.753
Diabetes mellitus	57 (32.9)	9 (27.3)	48 (34.3)	0.572
Cardiovascular disease	34 (19.7)	6 (18.2)	28 (20.0)	>0.999
Cerebrovascular disease	48 (27.7)	9 (27.3)	39 (27.9)	>0.999
Chronic kidney disease	25 (14.5)	2 (6.1)	23 (16.4)	0.212
Dialysis-dependent	11 (6.4)	1 (3.0)	10 (7.1)	0.635
Chronic liver disease	10 (5.8)	1 (3.0)	9 (6.4)	0.735
Connective tissue disease	3 (1.7)	1 (3.0)	2 (1.4)	>0.999
Cancer	19 (11.0)	2 (6.1)	17 (12.1)	0.487
Immunosuppression	20 (11.6)	2 (6.1)	18 (12.9)	0.426
ICU type				0.355
Medical ICU	124 (71.7)	21 (63.6)	103 (73.6)	
Surgical ICU	49 (28.3)	12 (36.4)	37 (26.4)	
On ICU admission				
APACHE II	15 (11–21)	14 (10–18)	17 (12–21)	0.085
SOFA	8 (4–10)	6 (4–9)	8 (5–11)	0.085
Vasopressor	100 (57.8)	17 (51.5)	83 (59.3)	0.537
Bacteremia	13 (7.5)	0 (0)	13 (9.3)	0.146
HFNC	16 (9.2)	1 (3.0)	15 (10.7)	0.300
Mechanical ventilation	111 (64.2)	22 (66.7)	89 (63.6)	0.895

Values are presented as median (interquartile range) for continuous variables and number (%) for categorical variables. ICU, intensive care unit; APACHE, Acute Physiological and Chronic Health Evaluation; SOFA, Sequential Organ Failure Assessment; HFNC, high-flow nasal cannula.

**Table 2:** Initial laboratory findings.

Variable	*N*	Total	Survivor (*n* = 33)	Non-Survivor (*n* = 140)	*p*-Value
White blood cells, 10^3^/L	173	10.5 (7.3–15.2)	10.8 (8.1–15.6)	10.4 (6.9–15.1)	0.868
Neutrophil–Lymphocyte ratio	169	9.0 (3.4–20.4)	5.9 (2.5–11.6)	9.7 (3.7–22.7)	0.153
Hematocrit, %	173	34.6 (29.5–39.0)	37.1 (31.2–41.1)	34.5 (29.4–38.5)	0.132
C-reactive protein, mg/dL	173	5.0 (0.3–17.5)	0.8 (0.1–3.2)	7.5 (0.4–18.4)	0.004
Albumin, g/dL	173	3.1 (2.6–3.8)	3.5 (3.2–4.0)	3.0 (2.6–3.6)	0.001
Bilirubin, mg/dL	173	0.6 (0.4–1.0)	0.5 (0.4–0.7)	0.6 (0.4–1.1)	0.184
Creatinine, mg/dL	173	1.0 (0.8–1.6)	1.0 (0.8–1.3)	1.0 (0.8–1.8)	0.489
Procalcitonin, mmol/L	106	1.2 (0.3–7.9)	0.5 (0.3–3.6)	1.2 (0.3–7.9)	0.538
Lactic acid, mmol/L	147	2.8 (1.7–5.0)	2.5 (1.2–4.7)	2.8 (1.8–5.0)	0.149

Values are presented as median (interquartile range). Denominators vary across laboratory variables due to missing data; variable-specific *N* is shown.

**Table 3:** Variables related to Carbapenem-resistant *Acinetobacter baumannii* infection and treatment details.

Variable	Total (*n* = 173)	Survivor (*n* = 33)	Non-Survivor (*n* = 140)	*p*-Value
Ventilator associated pneumonia	132 (76.3)	23 (69.7)	109 (77.9)	0.445
SOFA ^a^	7 (5–10)	6 (4–7)	8 (5–10)	0.001
CRAB bacteremia	26 (15.0)	1 (3.0)	25 (17.9)	0.061
CRAB from other sterile sites ^b^	11 (6.4)	2 (6.1)	9 (6.4)	>0.999
Polymicrobial infection	50 (28.9)	8 (24.2)	42 (30.0)	0.658
Medications				
Corticosteroid	107 (61.8)	14 (42.4)	93 (66.4)	0.019
Carbapenem	144 (83.2)	26 (78.8)	118 (84.3)	0.616
Minocycline	82 (47.4)	21 (63.6)	61 (43.6)	0.060
Colistin (intravenous)	34 (19.7)	8 (24.2)	26 (18.6)	0.621
Colistin (inhalation)	35 (20.0)	6 (18.2)	29 (20.7)	0.932
Ampicillin–sulbactam	3 (1.7)	1 (3.0)	2 (1.4)	>0.999
Aminoglycoside	19 (11.0)	2 (6.1)	17 (12.1)	0.487
Combination therapy	100 (57.8)	17 (51.5)	83 (59.3)	0.537
ICU interventions				
Mechanical ventilation	164 (94.8)	30 (90.9)	134 (95.7)	0.495
Initiation of renal replacement therapy	41 (23.7)	2 (6.1)	39 (27.9)	0.015
Re-intubation	24 (13.9)	4 (12.1)	20 (14.3)	0.965
Pleural drainage	43 (24.9)	9 (27.3)	34 (24.3)	0.894
ECMO	4 (2.3)	0 (0)	4 (2.9)	0.735

Values are presented as median (interquartile range) for continuous variables and number (%) for categorical variables. SOFA, Sequential Organ Failure Assessment; CRAB, carbapenem-resistant *Acinetobacter baumannii*; ICU, intensive care unit; ECMO, extracorporeal membrane oxygenation. ^a^ on the day of the index culture collection. ^b^ such as pleural, abdominal, and cerebrospinal fluids.

**Table 4:** Factors associated with in-hospital death identified through multivariable logistic regression analysis.

Variable	Univariate Analysis			Multivariable Analysis		
	OR	95% CI	*p*-Value	OR	95% CI	*p*-Value
Age	1.02	0.99–1.05	0.125			
APACHE II	1.06	1.00–1.13	0.072			
C-reactive protein	1.06	1.01–1.12	0.018			
Albumin	0.44	0.25–0.72	0.002	0.55	0.31–0.95	0.031
SOFA ^a^	1.23	1.08–1.42	0.003	1.15	0.99–1.33	0.065
Corticosteroid use	2.69	1.25–5.92	0.012	2.82	1.23–6.50	0.015
Minocycline use	0.44	0.20–0.95	0.041			
Initiation of renal replacement therapy	5.99	1.70–38.1	0.018	4.40	0.93–20.74	0.061

OR, odds ratio; CI, confidence interval; APACHE, Acute Physiological and Chronic Health Evaluation; SOFA, Sequential Organ Failure Assessment. ^a^ on the day of the index culture collection.

**Table 5:** Factors associated with clinical failure at day 14 identified through multivariable logistic regression analysis.

Variable	Univariate Analysis			Multivariable Analysis		
	OR	95% CI	*p*-Value	OR	95% CI	*p*-Value
Age	1.02	0.99–1.04	0.147	1.02	0.99–1.06	0.114
ICU type—Surgical ^a^	0.41	0.20–0.86	0.018	0.51	0.20–1.30	0.155
APACHE II	1.05	0.99–1.12	0.095			
C-reactive protein	1.09	1.04–1.15	<0.001	1.08	1.02–1.15	0.008
Albumin	0.51	0.31–0.80	0.005			
SOFA ^b^	1.26	1.12–1.44	<0.001	1.27	1.10–1.46	0.001
Corticosteroid use	2.48	1.22–5.08	0.012	1.93	0.81–4.59	0.136
Minocycline use	0.23	0.10–0.48	<0.001	0.21	0.08–0.50	0.001
Combination therapy	0.46	0.21–0.95	0.043			

OR, odds ratio; CI, confidence interval; ICU, intensive care unit; APACHE, Acute Physiological and Chronic Health Evaluation; SOFA, Sequential Organ Failure Assessment. ^a^ Medical ICU was used as the reference category. ^b^ on the day of the index culture collection.

## Data Availability

The data presented in this study are available on request from the corresponding author. The data are not publicly available due to privacy or ethical restrictions.

## Supplementary Materials

The following supporting information can be downloaded at https://www.mdpi.com/article/10.3390/jcm15103594/s1, Table S1. Multivariable logistic regression analysis of factors associated with in-hospital death, including patients with pre-existing do-not-resuscitate orders; Table S2. Subgroup multivariable logistic regression analysis of factors associated with in-hospital death among patients receiving corticosteroids (*n* = 107).

## Abbreviations

APACHE II = Acute Physiology and Chronic Health Evaluation II, CRAB = carbapenem-resistant *Acinetobacter baumannii*, CRP = C-reactive protein, DNR = do-not-resuscitate, ICU = intensive care unit, RRT = renal replacement therapy, SOFA = Sequential Organ Failure Assessment, VAP = ventilator-associated pneumonia.
