# Supplementary material for: Factors Associated with Short-Term Clinical Outcomes in ICU Patients with Carbapenem-Resistant Acinetobacter baumannii Pneumonia: A Single-Center Retrospective Cohort Study
Source: J Clin Med. 2026 May 8;15(10):3594. doi: 10.3390/jcm15103594 (PMC13208023; doi:10.3390/jcm15103594)
Supplement: Supplementary file 1 [file jcm-15-03594-s001.zip › jcm-4245237-supplementary.pdf]

Supplementary Table S1. Multivariable logistic regression analysis of factors associated with in-hospital death, including patients with pre-existing do-not-resuscitate orders

	OR	95% CI	P-value
Albumin	0.55	0.32–0.95	0.030
SOFA [a]	1.14	0.99–1.32	0.065
Corticosteroid use	2.60	1.16–5.83	0.021
Initiation of renal replacement therapy	2.73	0.73–10.23	0.136

OR, odds ratio; CI, confidence interval; SOFA, Sequential Organ Failure Assessment.

[a] on the day of the index culture collection.

Supplementary Table S2. Subgroup multivariable logistic regression analysis of factors associated with in-hospital death among patients receiving corticosteroids (n = 107)

Variable	Univariate analysis			Multivariable analysis		
	OR	95% CI	P-value	OR	95% CI	P-value
Age	1.01	0.97–1.05	0.637			
APACHE II	1.21	1.08–1.39	0.003	1.14	0.98–1.32	0.082
C-reactive protein	1.05	1.00–1.14	0.120			
Albumin	0.25	0.10–0.55	0.001	0.33	0.11–1.02	0.054
SOFA [a]	1.40	1.14–1.80	0.003	1.22	0.95–1.55	0.114
Minocycline use	0.32	0.08–1.02	0.066	0.19	0.04–0.90	0.036
Prednisone-equivalent dose, per 10 mg/day increase	0.92	0.81–1.05	0.174			

OR, odds ratio; CI, confidence interval; APACHE, Acute Physiological and Chronic Health Evaluation; SOFA, Sequential Organ Failure Assessment.

Blank cells indicate variables entered into the initial multivariable model but not retained after backward elimination.

[a] on the day of the index culture collection.
